# Relationship between endocardial R-wave amplitude at the apical lead location and regional right ventricular strain analysis

**DOI:** 10.1590/1806-9282.20241621

**Published:** 2025-06-02

**Authors:** Ahmet Özderya, Fatih Gülçebi, Murat Gökhan Yerlikaya, Mehmet Ali Maz, Sinan Şahin, Muhammet Raşit Sayın

**Affiliations:** 1 Trabzon Kanuni Training and Research Hospital, Department of Cardiology – Trabzon, Turkey.; 2 University of Health Sciences, Trabzon Ahi Evren Thoracic and Cardiovascular Surgery Training and Research Hospital, Department of Cardiology – Trabzon, Turkey.; 3 University of Health Sciences, Şişli Hamidiye Etfal Training and Research Hospital, Department of Cardiology – Istanbul, Turkey.

**Keywords:** 2 D doppler echocardiography, Cardiac pacemakers, Cardioverter defibrillator, implantable

## Abstract

**OBJECTIVE:**

Cardiac implantable electronic devices are widely used today. Therefore, research is ongoing to provide better device implantation in technical terms. The aim of this study was to investigate the relationship between pre-procedural regional right ventricular strain and post-procedural endocardial R-wave amplitude in patients scheduled to receive an implantable cardiac defibrillator.

**METHODS:**

A total of 112 patients who underwent single-chamber implantable cardiac defibrillator implantation were included in the study. Right ventricular strain analysis was performed before the procedure, and the following parameters were recorded: four-chamber strain, free wall strain, septal strain, and apical strain. The relationship between R-wave amplitude, calculated after lead implantation in the apical region, and strain parameters was statistically analyzed.

**RESULTS:**

All strain parameters were statistically significantly better in the group with a high R-wave amplitude. Correlation analysis showed that a higher R-wave amplitude was associated with improved right ventricular four-chamber strain (p<0.001, correlation coefficient=0.436), right ventricular free wall strain (p<0.001, correlation coefficient=0.532), right ventricular septal strain (p<0.001, correlation coefficient=0.394), and right ventricular apical strain (p<0.001, correlation coefficient=0.814). In univariable regression analysis, all strain parameters were identified as dependent predictors; however, in multivariable regression analysis, only right ventricular apical strain (p<0.001) was found to be an independent predictor of high R-wave amplitude.

**CONCLUSION:**

Our study revealed a relationship between the right ventricular apical endocardial R-wave amplitude and all right ventricular strain parameters, especially right ventricular apical strain. We recommend that clinicians perform regional right ventricular strain analysis before implantable cardiac defibrillator implantation in cases where lead positioning is uncertain.

## INTRODUCTION

Cardiac implantable electronic devices (CIEDs) are frequently used today as permanent pacemakers for conditions, such as symptomatic bradycardia, atrioventricular blocks, recurrent syncope, or sick sinus syndrome. They are also employed as implantable cardiac defibrillators (ICDs) based on malignant arrhythmia risk assessment^1^. The most common site for ventricular lead placement during CIED implantation is the apical region of the right ventricle (RV)^2^. However, lead implantations have also been performed in various other anatomical locations within the RV, such as the outflow tract and the septum, as well as in specialized sites such as the bundle of His and the left bundle branch for pacing^3–5^.

During CIED implantations, measurements of R-wave amplitude, threshold, and impedance are crucial once the lead contacts the ventricle. For a lead position to be considered effective, the R-wave amplitude recorded from the ventricular electrogram should be ≥5 Mv^6^. In studies focusing on optimal ventricular lead placement, a high R-wave amplitude is regarded as an indicator of success^7^.

Echocardiography is frequently used both before CIED implantation and during follow-up. Strain analysis, a specific echocardiographic examination, is also employed in CIED follow-ups, according to the literature^8,9^. RV strain analysis was standardized in a 2018 review by Badano et al., providing researchers with the opportunity to evaluate RV strain regionally^10^.

In this study, we aimed to investigate the relationship between pre-procedural RV regional strain parameters and post-procedural endocardial R-wave amplitude in patients undergoing CIED implantation.

## METHODS

### Study design and population

This single-center, prospective study included patients who underwent single-chamber implantable cardiac defibrillator (VVI-ICD) implantation in our clinic between April 2023 and April 2024. To minimize variability between ICD devices, only patients who received the Boston Scientific VVI-ICD (the most commonly used model in our clinic) were considered eligible for the study. Patients implanted with other VVI-ICD makes and models were excluded from the study. Furthermore, only patients who had the lead implanted in the RV apical region were considered, while those with lead implantations in the septal region were excluded to avoid inconsistencies. Additionally, eight patients were excluded from the study due to imaging limitations that prevented strain analysis before the procedure. After applying the exclusion criteria, 112 patients were included in the sample, and their demographic data and medical histories were recorded.

### Ethical considerations

The study protocol was approved by the local ethics committee in accordance with the Declaration of Helsinki and Good Clinical Practice guidelines.

### Laboratory and echocardiographic evaluation

Blood samples were collected from peripheral veins upon the patients’ first hospital admission, and tests were performed for complete blood count, fasting blood glucose, kidney and liver function, lipid panel, C-reactive protein (CRP), total protein, and albumin. A complete blood count was measured using the Mindray BC-5800 automatic hematology analyzer (Mindray Medical Electronics Co., Shenzhen, China). All patient data were obtained and recorded through the hospital database.

Patients were transferred to the echocardiography laboratory for examination. After achieving optimal conditions for blood pressure measurement, systolic and diastolic blood pressures were recorded. An echocardiographic examination was performed using the Philips EpiQ-7 system (X5 probe, Philips^®^ Medical Systems, Andover, MA). Left ventricular ejection fraction was calculated using Simpson's method. From the parasternal long-axis view, end-diastolic diameter, end-systolic diameter, interventricular septum, and posterior wall (PW) were measured. The right atrium and RV were measured in the apical four-chamber view. Systolic pulmonary artery pressure was calculated by adding tricuspid peak velocity to the estimated right atrial pressure.

**Figure 1:**
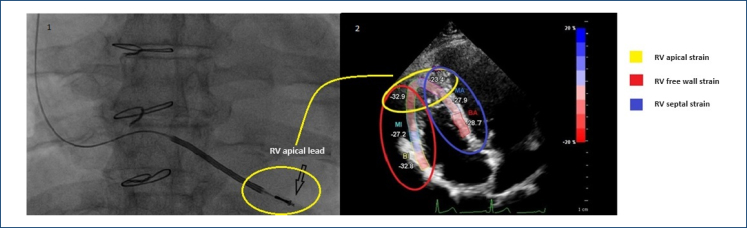
Demonstration of lead positioning in the right ventricular apical region on fluoroscopy with echocardiographic apical strain diagram.

Two-dimensional (2D) speckle tracking echocardiography was performed using CMQ software (QLAB 10.3; Philips Medical System, Andover, MA) to evaluate strain imaging. For RV strain analysis, the RV boundaries were initially identified automatically using an RV-focused apical four-chamber view, followed by manual corrections. RV free wall strain was calculated as the average of the three segments on the free wall, while RV septal strain was calculated as the average of the three segments on the septum. For RV four-chamber strain was recorded as the average of all six segments (three free wall and three septum measurements)^10^. Since the RV apex was chosen as the lead implantation site in our study (Figure 1) and strain analysis provided the opportunity to perform regional analysis, we defined a new parameter called "RV apical strain," which we determined as the average of the two apical segments in the septal and free wall regions (Figure 2). All echocardiograms were obtained and interpreted by two experienced cardiologists blinded to the patients’ conditions, following the recommendations of the American Society of Echocardiography^11^.

### Cardiac implantable electronic device implantation and measurement of R-wave amplitude

The patients underwent venous puncture via the left subclavian vein. After the venous sheath was placed, the ventricular lead was advanced and implanted into the RV apex using a modified stylet (Figure 1). Following lead fixation, the endocardial R-wave amplitude was measured using the Boston 3300 Latitude Programming System. The R-wave amplitude was determined by averaging three consecutive stable measurements. After the measurements, the battery was implanted, and the process was completed by performing the necessary maneuvers.

### Statistical analysis

Statistical analyses were performed using SPSS version 20.0 for Windows (SPSS Inc., Chicago, IL, USA). The Kolmogorov-Smirnov test and homogeneity of variance test were performed to examine parametric and non-parametric data distributions, respectively. The independent-sample t-test was used to compare two groups in terms of variables with parametric distributions, while the Mann-Whitney U test was used for the two-group comparison of variables that did not show a parametric distribution. Categorical variables were compared using the chi-square test. Pearson's and Spearman's correlation analyses were undertaken to evaluate the relationship between the R-wave amplitude and strain parameters. Univariable and multivariable logistic regression analyses were performed to determine predictive parameters of high R-wave amplitude. A p-value of <0.05 was considered statistically significant.

## RESULTS

A total of 112 patients were included in the study. The R-wave median value was seen as 12 (minimum=5.6–maximum=25.1). Based on the median R-wave amplitude value, the patients were divided into two groups: the supramedian R-wave amplitude group (Group 1) and the inframedian R-wave amplitude group (Group 2). Group 1 consisted of 57 patients, while Group 2 comprised 55 patients. Of the 112 patients, 57 were implanted with ICD for primary prevention with a diagnosis of ischemic heart failure, 26 with non-ischemic heart failure, and 5 with hypertrophic cardiomyopathy. ICD implantation was performed in 24 patients for secondary prevention due to ventricular arrhythmia. The demographic characteristics, clinical features, and laboratory parameters of the groups are given in Table 1. No statistically significant differences were observed between the two groups in any of these parameters.

**Table 1:** Clinical characteristics and laboratory and echocardiography parameters of the study population.

	Supramedian R-wave amplitude	Inframedian R-wave amplitude	p-value
Age (years)	56.23±13.63	60.12±12.48	0.118
Gender (F/M) (n)	50/7	41/14	0.092
BMI (kg/m^2^)	27.76±3.31	27.2±3.53	0.385
Ischemic CHF (n)	30	27	0.457
Non-ischemic CHF (n)	12	14	0.824
HCMP (n)	3	2	0.676
Ventricular arrhythmia (n)	12	12	0.950
Rhythm (SR/AF)	54/3	51/4	0.990
Pulse rate (beats/minute)	70.12±9.23	73.29±11.26	0.107
SBP (mmHg)	125 (100–150)	120 (100–150)	0.924
DBP (mmHg)	80 (60–90)	80 (60–95)	0.407
Glucose (mg/dL)	98 (69–380)	112 (64–390)	0.374
Creatine (mg/dL)	0.94±0.29	1.03±0.4	0.172
CRP (mg/L)	1 (0.1–70)	1 (0.1–65)	0.619
Total cholesterol (mg/dL)	152.88±43.2	148.94±33.68	0.595
LDL (mg/dL)	97.9±39.16	92.57±31.16	0.432
Hemoglobin (g/dL)	14.17±1.75	13.88±1.32	0.067
White blood cell count (×10^9^/L)	7.6 (5.02–20.8)	7.7 (5.1–19.9)	0.643
Platelet count (×10^9^/L)	215.3±69.65	204.77±59.62	0.391
LV-EF (%)	30 (15–60)	30 (15–60)	0.159
LV-EDD (mm)	60.8±7.3	59.71±7.1	0.426
LV-ESD (mm)	48.07±8.84	47.59±8.65	0.774
RV (mm)	34.29±4.52	34.19±4.88	0.913
TAPSE (mm)	21.92±3.93	20.94±4.92	0.248
S’ (mm)	14 (7–18)	13 (7–18)	**0.042**
sPAP (mmHg)	22 (18–65)	22 (17–48)	0.617
RV four-chamber strain	16.73±4.03	13.54±4.79	**0.001**
RV free wall strain	22.82±4.47	18.9±4.61	**<0.001**
RV septal strain	15.72±3.21	12.89±3.63	**<0.001**
RV apical strain	24.09±4.93	15.02±6.72	**<0.001**

AF: atrial fibrillation; BMI: body mass index; CKD: chronic kidney disease; CRP: C-reactive protein; HCMP: hypertrophic cardiomyopathy; LDL: low-density lipoprotein; LV-EDD: left ventricular end-diastolic diameter; LV-EF: left ventricular ejection fraction; LV-ESC: left ventricular end-systolic diameter; RV: right ventricle; SR: sinus rhythm; S’: tricuspid lateral wall annular systolic velocity; sPAP: systolic pulmonary arterial pressure; TAPSE: tricuspid annular plane systolic excursion; SBP: systolic blood pressure; DBP: diastolic blood pressure; CHF: congestive heart failure. Statistically significant values are denoted in bold.

Table 1 presents the comparison of the echocardiographic parameters of the groups. There were statistically significant differences regarding S wave (p=0.042), RV four-chamber strain (p=0.001), RV free wall strain (p<0.001), RV septal strain (p<0.001), or RV apical strain (p<0.001). When the correlation analysis between strain parameters and the R-wave amplitude was examined, statistically significant differences regarding RV four-chamber strain (p<0.001, correlation coefficient [CC]=0.436), RV free wall strain (p<0.001, CC=0.532), RV septal strain (p<0.001, CC=0.394), and RV apical strain (p<0.001, CC=0.814) were seen.

Table 2 shows the results of the univariable regression analysis performed on all parameters to identify predictors of high R-wave amplitude. Significant parameters from the univariable analysis were included in a multivariable regression analysis.

**Table 2:** Univariable and multivariable regression analyses showing the relationship between high R-wave amplitude and strain parameters.

Variables	Univariable analysis			Multivariable analysis		
	OR	95%CI	p	OR	95%CI	p
RV four-chamber strain	1.177	1.064–1.302	**0.002**	0.758	0.571–1.007	0.056
RV free wall strain	1.206	1.089–1.335	**<0.001**	1.010	0.821–1.242	0.928
RV septal strain	1.270	1.112–1.452	**<0.001**	1.061	0.769–1.464	0.718
RV apical strain	1.250	1.155–1.353	**<0.001**	1.374	1.156–1.635	**<0.001**

OR: odds ratio; CI: confidence interval; RV: right ventricle. Statistically significant values are denoted in bold.

## DISCUSSION

This study explored the relationship between pre-procedural echocardiographic regional strain parameters and post-procedural endocardial R-wave amplitude in patients undergoing VVI-ICD implantation. The study included 112 patients with ischemic heart failure, non-ischemic heart failure, or no heart failure, who received VVI-ICDs for primary or secondary prevention. By including patients with varying ejection fractions, we aimed to obtain a broad range of ventricular strain values. To maintain homogeneity and eliminate potential device- or lead-related variability, only patients receiving the same type of battery and lead were included in the study. In addition, to ensure consistency, all procedures were performed by the same experienced surgical team in a clinic performing an average of around 600 CIED implantations annually.

Our study focused exclusively on lead implantations in the RV apical region. While the RV apical region has been the most frequently used site for lead implantation, it has recently fallen out of favor due to its incompatibility with the physiological conduction system and the frequent occurrence of complications, such as perforation^12^. However, the RV apex remains one of the most frequently used sites for lead implantation based on both literature and operator experience.

Apart from electrophysiological pacing techniques such as the bundle of His and the left bundle branch for pacing, comparisons between RV apex and RV septal lead placements have been debated. Although RV apex implantation is often considered less physiological and is thought to impair cardiac function in the long term, Galand et al. published a study showing that RV apical pacing did not have a more negative effect on cardiac function compared to septal pacing^4^. Similarly, in a study by Garg et al.^13^ no significant differences were found between apical and non-apical ICD implantations in terms of mortality, inappropriate shocks, or shock success. This may explain why implanting leads in the apical region of the RV continues to be practiced. While the "2021 ESC Guidelines on Cardiac Pacing and Cardiac Resynchronization Therapy" do not impose strict restrictions on lead implantation sites, they recommend making patient-specific decisions^14^. While reviewing the published studies and guidelines, it becomes evident that there is still a gap in evidence regarding the optimal positioning of leads. This led us to conduct the current study to answer the question of whether echocardiographic analysis of the target area can provide insights into its suitability for lead implantation before the ICD procedure.

In our study, we selected patients with RV apical lead implantation to ensure both a high level of operator experience and more stable lead positioning. We used strain analysis in the echocardiographic evaluation. Strain analysis has been extensively used in RV studies, and its effectiveness has been well established^15,16^. In the literature, RV strain analysis is typically divided into six segments: septum basal, septum mid, septum apical, free wall basal, free wall mid, and free wall apical segments^10,15^. During the statistical analysis of our data, we observed that strain values from the apical region were highly correlated with the R-wave amplitude. Based on this, we introduced a new parameter called "RV apical strain," calculated as the average of the apical septum and the apical free wall measurements, which has not been previously defined in the literature.

Our results, as presented in Table 1, indicate that better strain values were achieved in the group with a higher R-wave amplitude. When we examined the correlation between RV strain parameters and the R-wave amplitude, the correlation coefficient between RV apical strain and the R-wave amplitude was 0.814, which is a remarkable result for both our study and the existing literature. In the regression analysis presented in Table 2, RV apical strain emerged as the only independent predictor among all strain parameters. This finding suggests that RV apical strain could assist clinicians when they are uncertain about the optimal region for lead placement during implantation.

## CONCLUSION

This study revealed a significant relationship between the endocardial R-wave amplitude recorded from the RV apical region and echocardiographic RV strain parameters. In particular, the high correlation between RV apical strain and the R-wave amplitude, as well as the identification of the former as an independent predictor of the latter, is a promising finding. We demonstrated that clinicians could potentially make better lead placement decisions by using regional strain analysis before the procedure in cases of uncertainty regarding lead positioning.

## LIMITATIONS

İt is acknowledged that 2D echocardiographic examinations and fluoroscopy evaluations have limitations in accurately identifying the true apex of the RV.
